# Relative positioning of Kv11.1 (hERG) K^+^ channel cytoplasmic domain-located fluorescent tags toward the plasma membrane

**DOI:** 10.1038/s41598-018-33492-x

**Published:** 2018-10-19

**Authors:** Francisco Barros, Pedro Domínguez, Pilar de la Peña

**Affiliations:** Departamento de Bioquímica y Biología Molecular, Universidad de Oviedo, Edificio Santiago Gascón, Campus de El Cristo, 33006 Oviedo, Asturias Spain

## Abstract

Recent cryo-EM data have provided a view of the KCNH potassium channels molecular structures. However, some details about the cytoplasmic domains organization and specially their rearrangements associated to channel functionality are still lacking. Here we used the voltage-dependent dipicrylamine (DPA)-induced quench of fluorescent proteins (FPS) linked to different positions at the cytoplasmic domains of KCNH2 (hERG) to gain some insights about the coarse structure of these channel parts. Fast voltage-clamp fluorometry with HEK293 cells expressing membrane-anchored FPs under conditions in which only the plasma membrane potential is modified, demonstrated DPA voltage-dependent translocation and subsequent FRET-triggered FP quenching. Our data demonstrate for the first time that the distance between an amino-terminal FP tag and the intracellular plasma membrane surface is shorter than that between the membrane and a C-terminally-located tag. The distances varied when the FPs were attached to other positions along the channel cytoplasmic domains. In some cases, we also detected slower fluorometric responses following the fast voltage-dependent dye translocation, indicating subsequent label movements orthogonal to the plasma membrane. This finding suggests the existence of additional conformational rearrangements in the hERG cytoplasmic domains, although their association with specific aspects of channel operation remains to be established.

## Introduction

Kv11.1 (hERG, *KCNH2*) K^+^ channels are expressed in a variety of non-cardiac cells in which they play a key role in setting their electrical behaviour^1–4^. Additionally, hERG channels mediate the cardiac I_Kr_ current that acts as an important determinant of action potential repolarization in the human ventricle and of pacemaking activity in heart nodes^2,5–7^. Thus, impairment of hERG function by mutations in the *KCNH2* gene or by a variety of drugs prolong the QT interval of electrocardiograms leading to inherited and acquired type 2 long-QT syndrome, increasing the risk of torsade de pointes arrhythmia, ventricular fibrillation and sudden cardiac death^2,8^.

**Figure 1:**
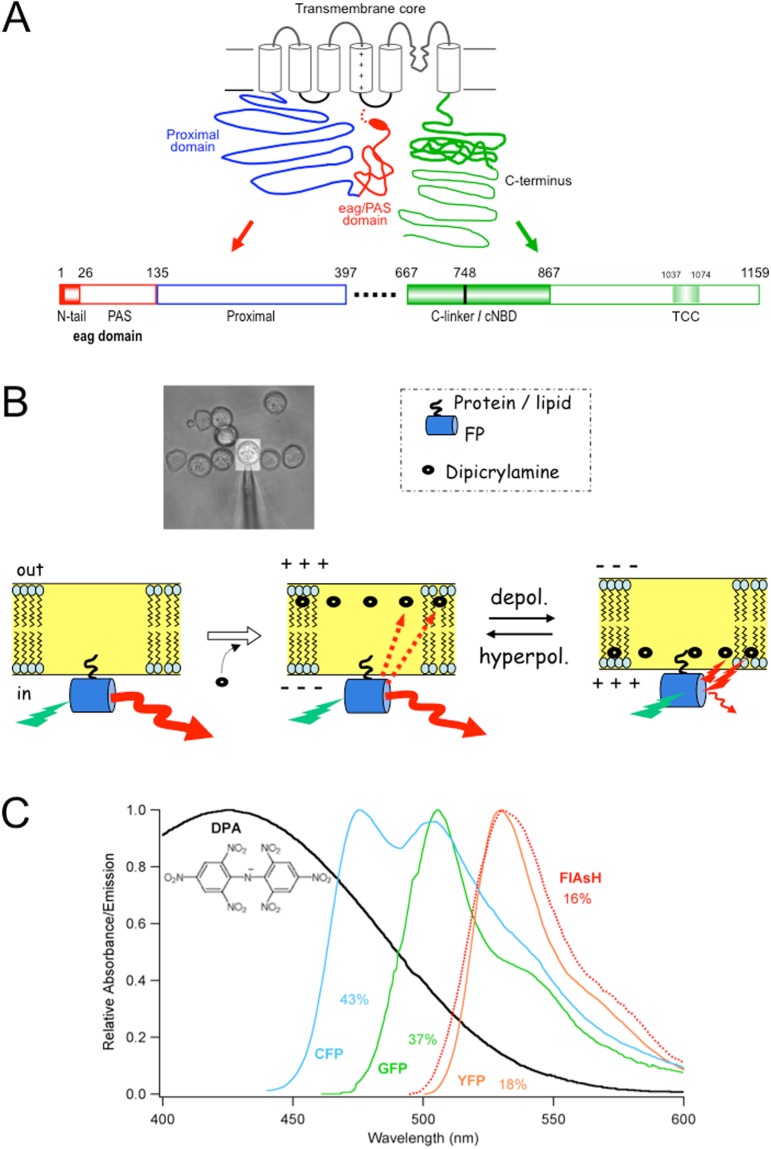
General topology of a hERG channel α-subunit and schematics of the DPA-based voltage-dependent FRET measurements. (**A**) Schematic representation of a hERG channel α-subunit indicating the relative positioning of the amino terminal *eag*/PAS and proximal domains up to the first transmembrane helix, and the C-terminus regions following the sixth transmembrane helix. The N-tail region including an initial flexible segment (dotted line) and the amphipatic α-helix (oval) connecting it to the Per-Arnt-sim (PAS) sub-domain (continuous line) of the *eag* domain, are depicted in red. The C-terminus is colored green. Linear diagrams of the amino and carboxy termini are shown at the bottom. The size of every segment is represented on a horizontal scale proportional to the total length of the fragments. The numbers correspond to the residues of the hERG sequence marking the boundaries of the different regions. The position of the C-linker/cNBD domains directly linked to the bottom of helix S6 and the location of a proposed tetramerization coiled-coil (TCC) are also shown at the C-terminus. The transmembrane core region containing the six transmembrane helices depicted as cylinders and the corresponding linkers are represented in black. The approximate points of FP insertion used are signaled by asterisks in the upper scheme and placed in their respective position in the lower linear diagrams. (**B**) Schematic representation of voltage-dependent DPA movements and FRET interactions with membrane-anchored FP fluorophores. An illustration of reversible DPA (black ovals) translocation in response to membrane voltage variations and the corresponding modifications in the fluorescence of an FP (yellow barrel) tag anchored to the internal membrane surface are shown. Different arrow length and thickness are used to highlight the change in fluorescence due to DPA approaching the donor upon depolarization and subsequent decrease of FP fluorescence as a result of increased FRET. A transmission image (40x objective) of the microscope field with the HEK293 cells and recording pipette attached to the selected cell limited by the ViewFinder selection mask (see Methods) is shown in the upper inset. (**C**) Spectral overlap (%) of CFP, GFP, YFP and FlAsH emission and DPA absorption. The structure of the negatively charged amphiphatic DPA molecule is shown in the inset.

hERG belongs to the voltage-gated family of potassium channels (Kv)^9^ characterized by a molecular architecture in which each subunit contains a pore-loop transmembrane region with six transmembrane helices (S1-S6), that are assembled as a tetramer of four identical (or similar) subunits surrounding a central pore, to which different cytoplasmic modules have been added through evolution^1^. In the case of hERG, this organization has been confirmed recently by cryo-EM structural data, that has also provided some insights about the general organization of the cytoplasmic domains^10^. However, some aspects about the arrangement of the amino terminals in relation to the rest of the cytoplasmic domains, as well as their positioning with respect to the channel core, remain obscure. In fact, in the case of hERG the cytoplasmic regions account for around eighty percent of the channel protein, but a substantial fraction of them was omitted in order to obtain stable cryo-EM protein samples, and their structure is lacking in the reported model^10^. As schematized in Fig. 1A, the hERG transmembranal core is preceded by a long amino moiety in which a short flexible segment and an amphipatic α-helix (known as amino terminal N-tail or CAP domain) is followed by a Per-Arnt-sim (PAS) domain, constituting the KCNH conserved region named eag/PAS domain, that extends from amino acids 1 to 135 (see 1,2,11 for reviews). This region is connected to the first transmembranal helix by a long stretch of residues for which no structural information is available, typical of hERG and known as proximal domain. At the beginning of the carboxy terminus, a C-linker region directly attached to the C-terminal portion of transmembrane helix S6 is followed by a cyclic nucleotide-binding domain (cNBD) homologous to those encountered in cyclic nucleotide gated (CNG) and hyperpolarization-activated cyclic nucleotide-gated (HCN) channels. Finally, a distal C-terminal region is located following the C-linker/cNBD, that contains a conserved coiled-coil domain termed TCC^1,2,11^. Interestingly, a crucial role of the amino- and carboxy-terminal cytoplasmic domains in activation and deactivation gating of hERG has been recognized^1,2,11^, this being particularly significant for this channel that operates as an inward rectifier although it has the typical molecular arrangement of the depolarization-activated channels^1,2,6,12^. Indeed, such a kinetic behaviour is critical for normal cardiac repolarization and for setting electrical parameters in other cells expressing hERG (reviewed in 8,13,14). Therefore, unraveling the general organization of hERG cytoplasmic regions is important for understanding how this channel controls fundamental physiological events such as cardiac rhythm and contraction, hormone secretion, and tumour cell proliferation^8,13,14^.

In this report, we used fast voltage-dependent dipicrylamine (DPA)-induced quenching of fluorescent proteins tagged at the amino and/or the carboxy terminus of hERG, to gain some insights about the coarse structure of these channel domains. DPA is a low-molecular-weight non-fluorescent hydrophobic anion (Fig. 1B,C) absorbing in the blue region of the light visible spectrum, that can act as an effective Förster-resonance-energy-transfer (FRET) acceptor from a variety of donor fluorophores^15–19^. Indeed, DPA has a significant spectral overlap with the emission of cyan, green and yellow fluorescent proteins, allowing for substantial energy transfer from them when located in close proximity to the non-fluorescent absorber. Furthermore, DPA partitions into the lipid membrane (e.g. the cell plasma membrane) close to the lipid-water interface, switching between the outer and inner membrane leaflets in a voltage-dependent way^15–23^. Thus, at internal membrane potentials of enough negative charge, the negatively charged DPA molecules almost exclusively populate the outward leaflet. Following a change in membrane potential, the redistribution of the DPA molecules across the plasma membrane is very rapid (typically one to a few ms), leading to a rapid alteration in fluorescence intensity of any donor associated with or closely located at only one of the membrane surfaces. This has been used to develop hybrid voltage sensors based in a combination of DPA with a FP or other FRET donors, to report fast membrane voltage changes such as action potentials in several cells^17,18,20–22^. Also, as shown schematically in Fig. 1B for the case of a fluorescent protein statically located respect to the internal membrane surface in such a short time scale, the application of a positive voltage step from a negative initial value will result in a lower fluorescence due to the increase in quenching derived from the very rapid movement of DPA closer to the position of the fluorophore. Since this rapid variation in the fluorometric signal is only due to the movement of the DPA probe, it is expected that the magnitude of the fluorescence change in response to a fixed magnitude voltage step can be used to determine the position of any static label with respect to the lipid bilayer acting as a reference plane. Both fast and steady-state measurements of DPA-induced quenching of different FRET donors have been used to map distances toward the membrane surface of tags labeling specific domains of some channels and pore-forming toxins^15,16,19,23,24^. We used here a similar strategy based in the voltage-dependent DPA translocation to demonstrate that the distance between a FP labeling the amino terminus of hERG and the intracellular plasma membrane surface is shorter than that between the membrane surface and a C-terminally-located tag. The N-terminal label distance to the membrane is also comparable to that between the membrane and a tandem construct carrying a CFP/YFP dimer directly anchored to the plasma membrane via a Rho prenylation signal (Rho-pYC). The distance to the membrane also varied when the attachment point of the FP was changed to other positions throughout the cytoplasmic domains of the channel. As also shown with CNGA1, TRPV1 and AMPAr channels^16,23,24^, we conclude that the observed variation in the quenching level due to changes in the position of the labels along the protein sequence and the additional detection of delayed fluorometric responses following the rapid DPA translocation, constitute a valuable method to gain some insights into the overall architecture of the hERG cytoplasmic domains, that could also allow the detection of conformational protein rearrangements associated with channel functions that cause orthogonal movements of the labels toward the plasma membrane.

## Results

### Voltage-dependent DPA quenching of membrane-anchored FP signals in a very fast time scale

**Figure 2:**
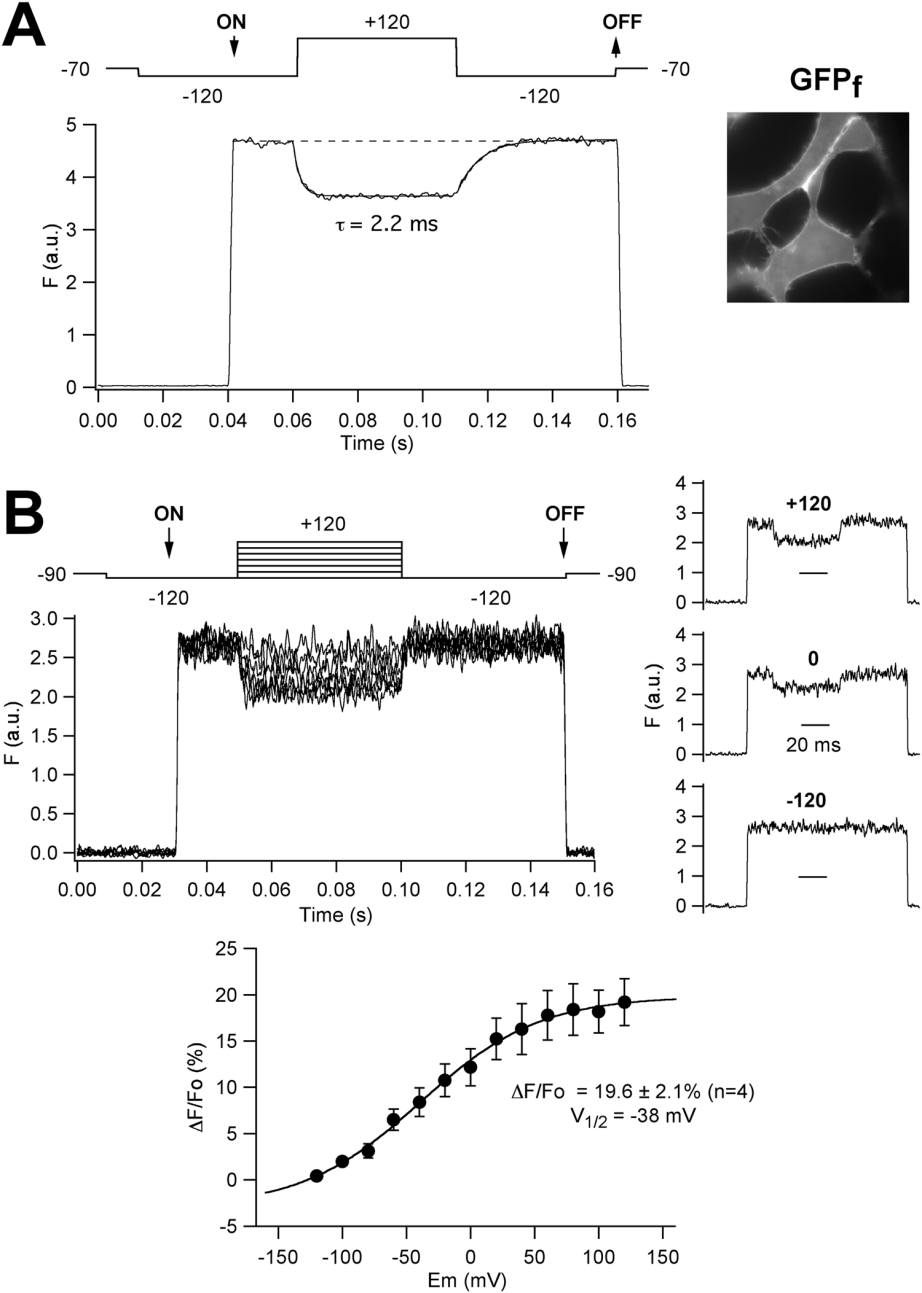
Voltage-dependent DPA-induced effects on patch-clamped HEK293 cells expressing plasma membrane-targeted green fluorescent protein (GFP_f_) carrying a farnesylation tag. (**A**) Time course of the fluorescent response on voltage pulses (shown on top of the traces) from −120 to +120 mV in the presence of 10 μM DPA. The time at which the monochromator wavelength was changed from 470 nm (corresponding to a zero volt input) to 498 nm and vice versa, is marked as ON and OFF, respectively. Note that no fluorescence is detected at 470 nm due to the cutoff properties of the filter set employed. For details, see Methods. Averaged responses (in arbitrary units) from 30 trials delivered at 1 s intervals are shown. The superimposed line corresponds to a mono-exponential fit to the data. A fluorescence image of the cells (100x oil-immersion objective) demonstrating the prominent plasma membrane distribution of the GFP_f_ signal is shown on the right. (**B**) Voltage dependence of the DPA-induced effects. A family of superimposed fluorescence traces in response to voltage steps as illustrated at the top including a central pulse from −120 to +120 mV at 10 mV intervals is shown on the upper left panel. Only results from pulses every 20 mV are shown on the graph for simplicity. For every voltage, traces correspond to averaged responses from 10 repetitive trials delivered at 1 s intervals. Separate traces obtained at +120, 0 and −120 mV are shown on the right. A plot of ΔF/Fo versus voltage, obtained from pulse responses such as those at the top, is shown at the bottom. Averaged responses from four individual cells are illustrated in the graph. A Boltzmann curve fitted to the data with V_1/2_ = −38 mV is also shown.

As an initial way to characterize the behavior of our DPA- and FP-based fluorometric system, we used a plasma membrane-targeted green fluorescent protein (GFP_f_) carrying a farnesylation tag^25^. As shown in Fig. 2A, application of a depolarizing voltage step from −120 to +120 mV to voltage-clamped HEK293 cells incubated in 10 μM DPA and expressing GFP_f_ (that is mostly anchored to the cytoplasmic face of the plasma membrane, see inset), induced a robust quenching of the FP fluorescence. This attenuation (expressed as ΔF/Fo values) averaged 16.3 ± 1.4% (n = 12) and developed in a very fast time scale, with a time constant of 3.1 ± 0.5 ms. Furthermore, the steady-state fluorescence *vs*. voltage relationship in response to voltage pulses of variable magnitude showed a sigmoidal shape with a V_1/2_ value of −38 ± 5 mV, n = 4 (Fig. 2B), an averaged ΔF/Fo of 19.6 ± 2.1% for the −120 to +120 mV jump, and a *z* value of 0.67 ± 0.13. Similar values have been previously reported by others^20^. The shape of the curve also indicated that whereas at negative voltages around −120 mV most of the DPA molecules are located near the outward membrane surface, the majority of them are near the inward plasma membrane surface at +120.

**Figure 3:**
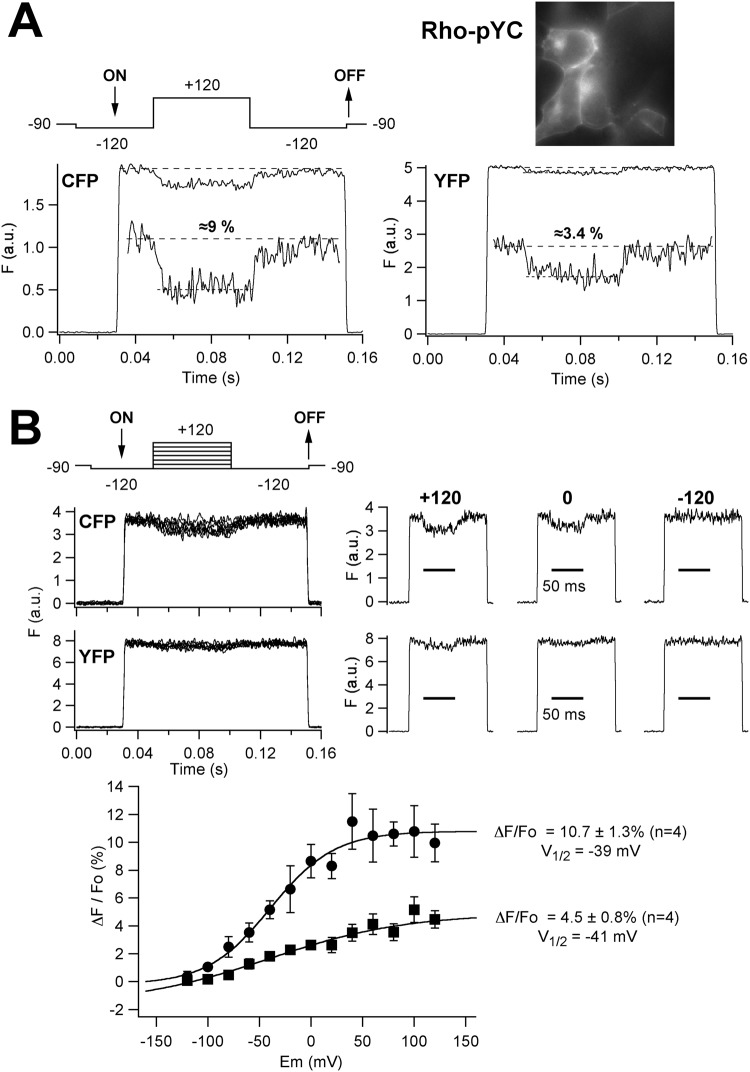
Voltage-dependent DPA-induced effects on patch-clamped HEK293 cells expressing the plasma membrane-targeted CFP-YFP Rho-pYC tandem. (**A**) Time course of the fluorescent response on voltage pulses from −120 to +120 mV (upper left panel) in the presence of 10 μM DPA. A fluorescence image of the cells (100x oil-immersion objective) demonstrating the marked plasma membrane distribution of the Rho-pYC signal is shown on the right. Representative fluorescence traces at excitation wavelengths of 440 (CFP) and 510 nm (YFP) are shown. Averaged responses from 50 trials delivered at 1 s intervals are illustrated on the graphs. Expanded sections of the fluorescence traces are shown in the insets. The estimated ΔF/Fo values expressed as % are also indicated. (**B**) Voltage dependence of the DPA-induced effects. Families of superimposed fluorescence traces in response to voltage steps as illustrated at the top are shown on the upper left panel. Separate traces obtained at +120, 0 and −120 mV are shown on the right. Plots of ΔF/Fo versus voltage with a Boltzmann curve fitted to the data, corresponding to the CFP (circles) and YFP (squares) signals, are shown at the bottom. V_1/2_ and maximal ΔF/Fo values obtained from the curves are indicated.

As indicated below, we used a collection of hERG channel constructs^26^ in which a cyan (CFP) and/or yellow (YFP) FP label was introduced at multiple positions throughout the hERG channel sequence. Therefore, we also characterized the voltage-dependent DPA-induced variation of FP fluorescence in cells expressing a YFP-CFP tandem (Rho-pYC)^27^ that is expected to be directly anchored to the plasma membrane via a Rho prenylation signal added to the C-terminal domain of a YFP-CFP fusion. Indeed, since the amino and carboxy ends of the FPs are quite close to one another and exit at the same extreme of their barrel structure^28^, it is expected that in this serial tandem the position of the two chromophores towards the plasma membrane would be similar. Therefore, this construct could also provide a better idea of the possible impact of the different spectral overlap of CFP and YFP with DPA on the quencher-induced variations of the FP signal. As in the case of GFP_f_, the FP fluorescence of the cells expressing Rho-pYC showed a clear peripheral pattern, indicating the preferential attachment of the dimer at the plasma membrane (Fig. 3A). Furthermore, the application of a voltage step from −120 to +120 mV to the Rho-pYC-expressing cells induced a fast depolarization- and a DPA-dependent quench of the FP fluorescence. As expected, when compared in the same cell, the magnitude of the attenuation was significantly higher for CFP (8.98 ± 1.3%, n = 6) than that of YFP (2.35 ± 0.32, n = 13). On the other hand, in both cases the DPA-induced response showed a sigmoidal voltage dependence with an equivalent V_1/2_ value (Fig. 3B), also analogous to that observed with GFP_f_ (see above). This reinforces the view that the fluorescence variations are caused by the translocation of DPA, and indicate that although the brightness of CFP in our fluorometric setup is clearly less than that of YFP, leading to a higher level of the overall recording noise, a more prominent quenching of the CFP signal is induced as a result of the more extensive spectral overlap of CFP and DPA (see Fig. 1B).

### DPA-induced quenching of FP-tagged hERG channel fluorescence

**Figure 4:**
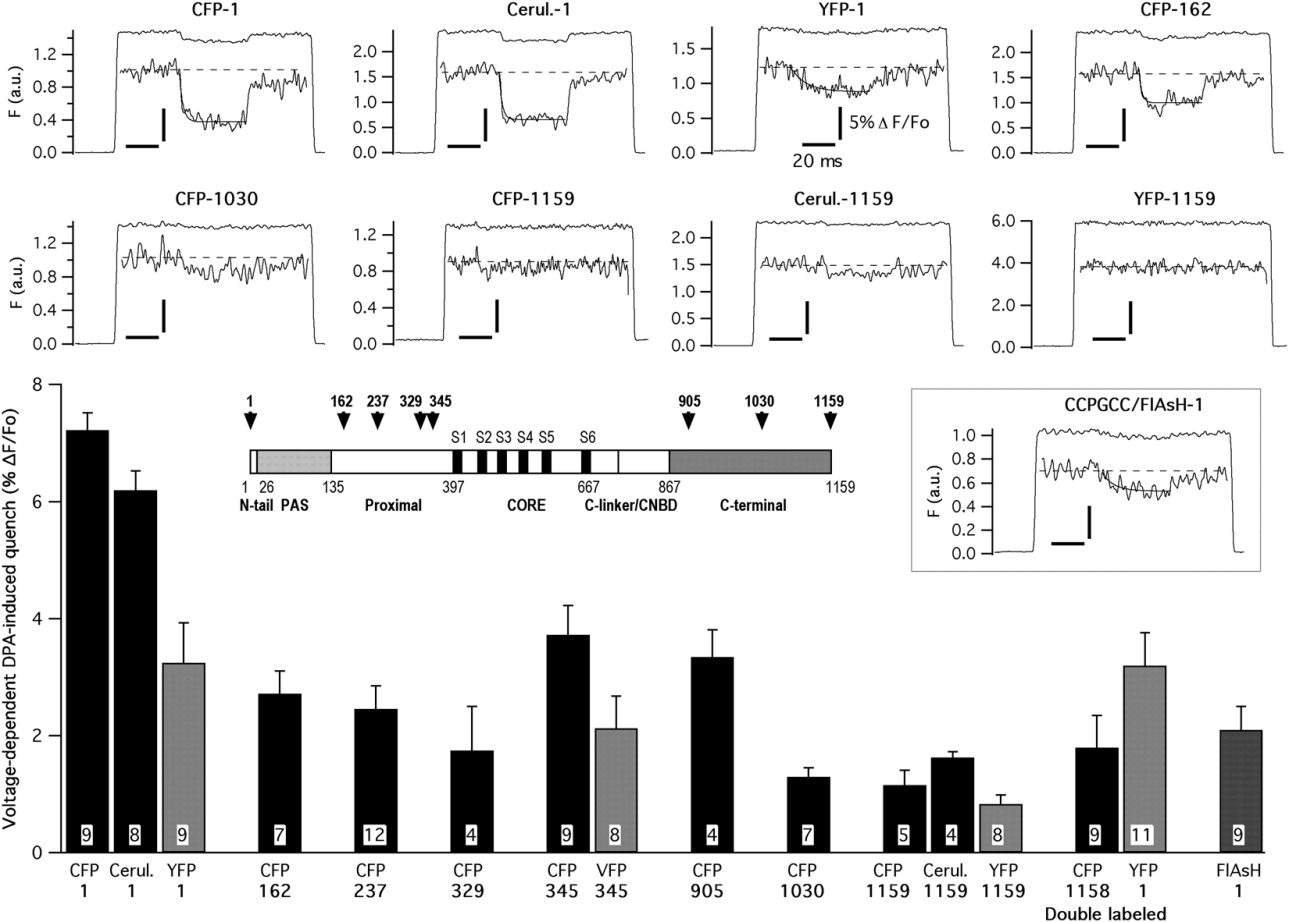
DPA voltage-dependent fluorescence quenching of hERG channels tagged with FPs at different positions. Representative fluorescence traces obtained in response to voltage step sequences such as those illustrated in Figs 1A and 2A, including a central pulse from −120 to +120 mV, are shown at the top. Data from patch-clamped HEK293 cells expressing hERG channels tagged with CFP, cerulean and YFP at the amino and carboxy ends, and with CFP at positions 162 and 1030 of the channel sequence are shown. Excitation wavelengths of 440 (CFP and cerulean) and 510 nm (YFP) were used. Averaged responses from 50 trials delivered at 1 s intervals are illustrated on the graphs. Expanded sections of the fluorescence traces are shown in the insets. In all cases, vertical and horizontal calibration bars correspond to 5% ΔF/Fo and 20 ms, respectively. Superimposed lines corresponding to mono-exponential fits to the data are shown on the upper file. Results from an analogous experiment using cells expressing a channel construct carrying an amino terminal CCPGCC sequence that was subsequently labeled with a FlAsH fluorophore (see text for details), are also shown. A summary of the DPA-dependent effects obtained with all the tested channel constructs is depicted at the bottom, indicating the number of tested cells and with the fluorophore and the point of insertion indicated below the bars of the histogram. A schematic linear diagram of the hERG channel in which the size of each domain is represented proportional to the total length of the protein is shown in the inset. The position of the boundaries between the different channel domains is marked with numbers. The places at which the fluorescent tags tested here were introduced are also indicated (arrows).

To look for possible differences in the distance between the tags situated at varying positions along the hERG channel sequence and the internal surface of the plasma membrane, we covalently attached cyan and yellow varieties of FPs at positions 1 and 1159 corresponding to the beginning and the end of the amino and the carboxy terminus, respectively (Fig. 4 inset). Additionally, CFP-containing constructs previously shown to yield functional channels^19^, carrying the fluorophore attached to positions 162, 237, 329 and 345 of the proximal domain in the amino terminus, and 905 and 1030 located in the last section of the carboxy terminus (Fig. 1C), were also tested for DPA-dependent fluorescent quenching. Four additional constructs carrying either YFP or the cyan variant cerulean at positions 1 and 1159, and one construct in which the Venus variant of YFP (VFP, a variant with almost identical spectral properties, a quantum yield slightly lower and an expected R_0_ value with DPA slightly smaller than those of EYFP)^29,30^ was introduced at position 345, were also used for further confirmation of the data. Constructs carrying FP labels at several positions of the amino terminal *eag*/PAS domain up to residue 135 and at the C-linker/cNBD region in the initial section (between residues 667 and 867) of the C-terminus were not tested due to their lack of functional expression^26^. As shown in Fig. 4, maximal quenching of fluorescence was attained with the amino terminus CFP-tagged construct (7.22 ± 0.3%, n = 9), followed (in decreasing magnitude of quench order) by constructs labeled with CFP at positions 345, 905, 162, 237, 329 and 1030, respectively. Interestingly, the smallest attenuations were observed with the distal C-tail CFP-1030 and the CFP-1159 constructs. When YFP/VFP instead CFP was the fluorophore linked to the protein at positions 1, 345 and 1159, the DPA-induced quenching was significantly smaller. As indicated above, this is consistent with the reduced spectral overlap of the yellow variants and DPA, as compared to that of CFP and the quencher. Also, the averaged effects of the cerulean constructs in the amino (Cerul.-1; 6.2 ± 0.32%, n = 8) and the carboxy terminus (Cerul.-1159; 1.63 ± 0.1%, n = 4) were equivalent to those observed with CFP, as expected from the almost identical spectral properties of both fluorescent proteins (Fig. 1B). The brightness of cerulean is higher than that of CFP^31^ leading to a better signal to noise ratio on the recordings, but only CFP-labeled channels in other positions of the hERG sequence were available^26^, such that we used CFP-based constructs for subsequent experiments. Finally, as additional support to the validity of the data obtained with the single labeled channel proteins, we also quantified the effect of DPA using a hERG channel construct double labeled (YFP-1 + CFP-1158) with YFP and CFP at the amino and carboxy terminus of the protein, respectively^26^. In this case, no significant differences were observed with respect to data obtained with the corresponding variants carrying either only YFP at position 1 or CFP at position 1159.

One possible concern about the data obtained using this method relates to the considerable volume of the FP-based constructs. Therefore, we also tested an additional channel variant, to which a tetracysteine sequence (CCPGCC) was added to the amino terminus, to be subsequently labeled with a YFP-like small FlAsH fluorescent moiety^32^ (see Methods) showing spectral characteristics almost identical to those of YFP (Fig. 1B). The CCPGCC-modified channels remained functional and showed almost unaltered activation properties (Suppl. Fig. 1). However, they appeared strongly accelerated in their deactivation kinetics, a property repeatedly recognized in other constructs carrying alterations at the tip of the N-terminus^26,27,33–35^. Indeed, this constitutes a further indication that the tetracysteine sequence was properly located at that position. Interestingly, the DPA-induced level of FlAsH tag fluorescence quenching (2.1 ± 0.4%, n = 9) showed no statistical differences with those observed with the single (3.25 ± 0.68%, n = 9) and double (3.2 ± 0.56%, n = 11) YFP-labeled channels at position 1 (Fig. 4). This indicates that in spite of the bigger molecular size of the FP-based tags, the distance of its central fluorophore toward the membrane reported by the DPA translocation appears to be similar to that exhibited by the considerably smaller tetracysteine/FlAsH-based constructs.

### Differences in DPA-induced FP quenching are not due to a different subcellular distribution of the hERG tagged channels

**Figure 5:**
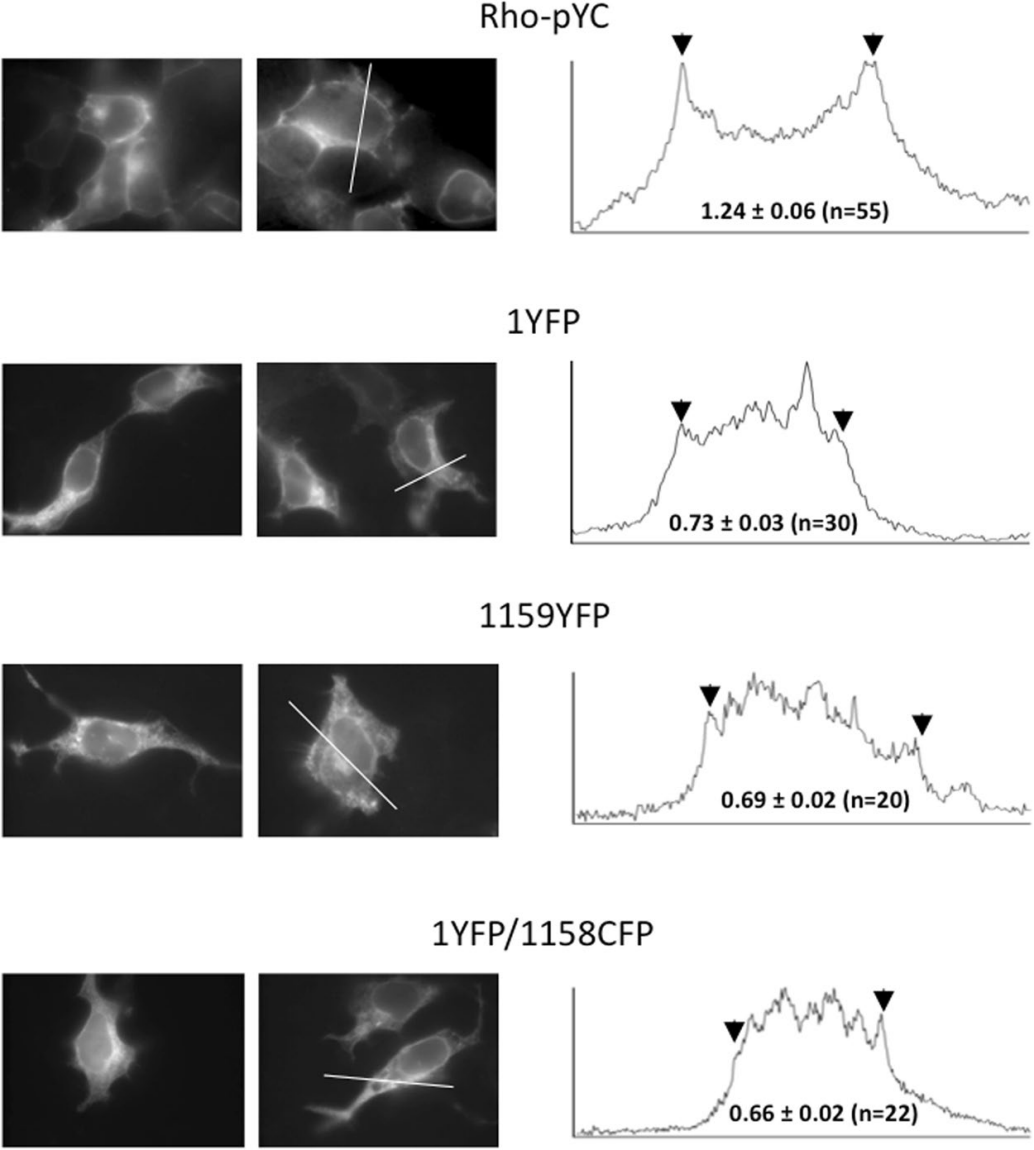
Cellular localization of hERG fluorescent fusion proteins carrying amino and carboxy terminal attached FPs. Representative cells expressing hERG-labeled channels with YFP at position 1 or 1159 or double-labeled 1YFP/1158CFP as indicated, and imaged by epifluorescence microscopy as described in Methods, are shown on the left. The top row corresponds to cells expressing the CFP-YFP Rho-pYC tandem. Fluorescence intensity profiles along the depicted white line are shown on the right. Arrows indicate the position of the fluorescence signal corresponding to the plasma membrane contour. Numbers in the profile panels indicate the values of the fluorescence intensity ratios obtained dividing the averaged fluorescence of the image pixels corresponding to a line traced on top of the cell edge (see Suppl. Fig. 2 for details) by the averaged fluorescence in the internal cell area.

One possible caveat when comparing the data obtained with different FP-labeled hERG constructs in the presence of DPA, in order to obtain conclusions about the relative positioning of the tags toward the plasma membrane surface, relates to the possibility that there could be a different surface-to-internal distribution of the protein. In fact, the ΔF factor constitutes a variation exclusively due to identical DPA transmembrane movement after a given voltage step, but it is necessary to ensure that the differences in the ΔF/Fo ratio obtained with the analyzed constructs are not due to the differential presence of labeled protein in internal membranes, that are not affected by the changes in membrane potential imposed by the plasma membrane-delimited voltage clamp. This could be particularly relevant in the case of hERG that, unlike the mostly plasma membrane located distribution observed with GFP_f_ and Rho-pYC, shows a diffuse fluorescent pattern throughout the cell, indicating a marked intracellular presence of the channel when expressed in HEK293 cells^26^ which could raise the level of overall Fo values. Apart from this being a cause of ΔF/Fo reductions, it will strongly limit the possibility of comparing the results obtained with the studied constructs unless their surface-to-internal distribution is similar. As exemplified in Fig. 5 for the two amino and carboxy end-labeled channel variants showing the most divergent DPA-induced responses, the subcellular location (see Methods) of the FP-labeled constructs demonstrated a marked intracellular distribution, as compared to the typical plasma membrane located distribution of Rho-pYC, characterized by a distinct fluorescent ring at the cell perimeter. However, the diffuse fluorescent pattern developed throughout the cell was similar for all channel constructs and, more importantly, in all cases they showed no significant differences when the F_contour_/F_internal_ ratio was quantitatively estimated (Fig. 5 and Suppl. Fig. 2). Therefore, even though some differences in the regional intracellular distribution of the different constructs cannot be discarded, the proportion of the intracellular *vs*. plasma membrane-derived fluorescence will be the same, indicating that the quantitative influence of the intracellularly located fluorescence is also the same. Thus, any variation in the voltage-dependent DPA-induced readout will be exclusively due to the different positioning of the labels toward the plasma membrane surface.

### Demonstration of slowly developing DPA-induced quenching in response to longer depolarizing steps

**Figure 6:**
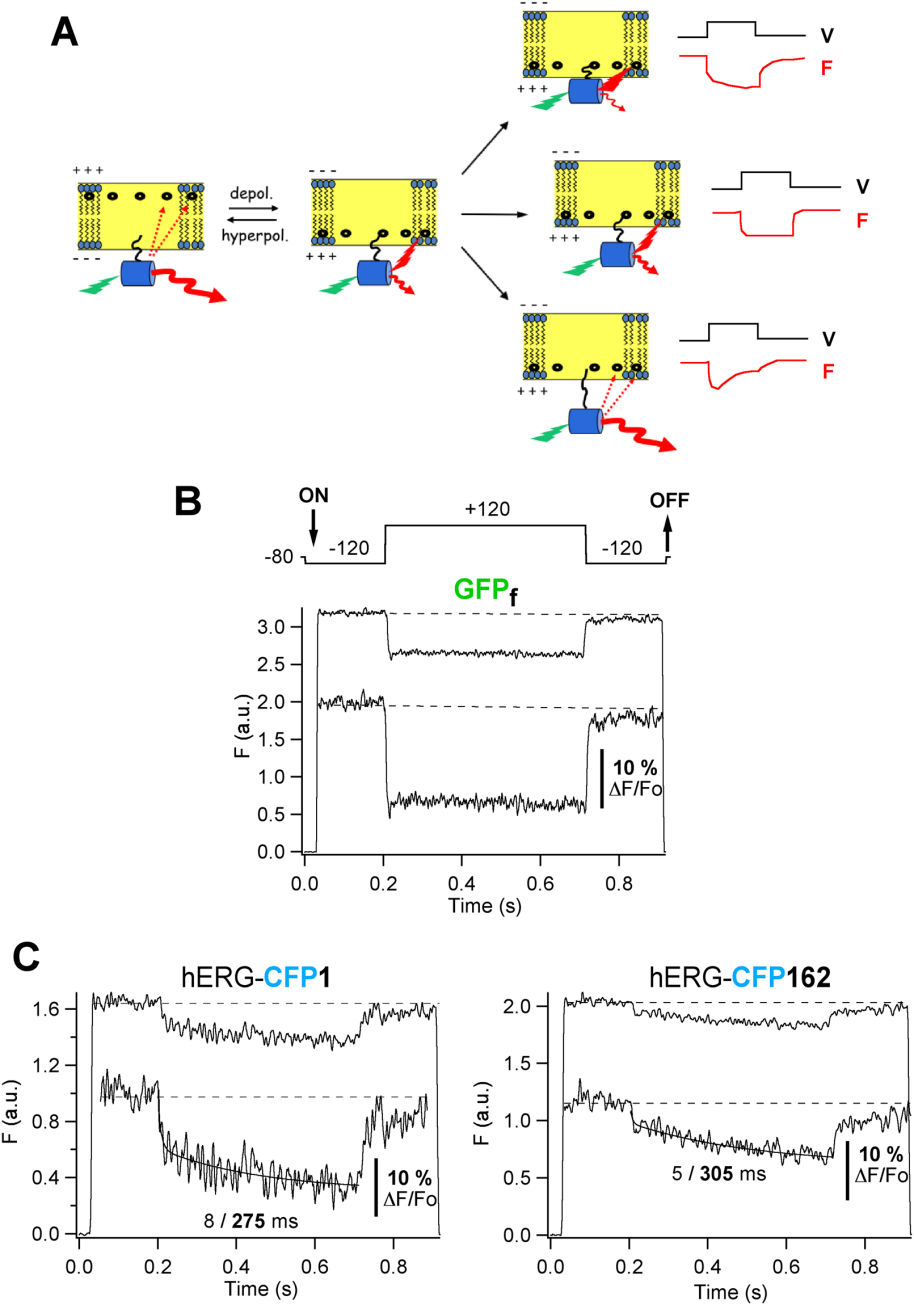
Detection of slowly developing DPA-induced quenching in response to longer depolarizing steps. (**A**) Schematic representation of possible secondary tag movements and hypothetical time course of fluorescence signals (F, red lines) in response to more prolonged depolarizing pulses (V, black lines), following the initial fast translocation of DPA. (**B**) Typical voltage-dependent DPA-induced response from cells expressing GFP_f_ in response to 500 ms depolarization steps as shown on top of the trace. Averaged responses from 50 trials delivered at 2 s intervals are shown. An expanded section of the fluorescence trace is shown in the inset. (**C**) Representative fluorometric trace from cells expressing hERG channels tagged with CFP at the amino end submitted to voltage-clamp steps as in panel A. A bi-exponential fit is shown superimposed to the data from which time constants of 8 and 275 ms were obtained as indicated. (**D**) Representative response from cells expressing hERG channels tagged with CFP at position 162. A bi-exponential fit with time constants of 5 and 305 ms is shown superimposed to the data.

As indicated above, the fast voltage- and time-dependent FP/donor fluorescence quenching triggered by DPA reflects the movement of the non-fluorescent acceptor normal to the plasma membrane plane^16,18,20,21,23^. This monotonic variation upon depolarization is only to be expected if an immobile fluorescent donor is located near the inner membrane leaflet and only DPA moves to it from the outer leaflet of the membrane bilayer (central row in Fig. 6A). Indeed, this is the behaviour observed when a 500 ms long depolarizing pulse was applied in the presence of DPA to cells expressing GFP_f_, that would act as a voltage-independent membrane-anchored immobile moiety (Fig. 6B). On the other hand, it is also expected that if a conformational rearrangement occurs in a FP-tagged protein carrier in response to the voltage step, a change in the distance of the fluorophore towards the membrane surface could be expected, and a secondary alteration in the fluorescence profile would take place. This could lead to either an additional decrease of donor fluorescence when it moves towards the membrane (Fig. 6A, upper row), or to a biphasic recovery of the signal when the fluorophore moves away from the membrane, which could increase the distance between it and the inner leaflet-located DPA and would decrease the FRET level between them (Fig. 6A, lower row). As shown in Fig. 6C, the application of a long depolarizing step to cells expressing channels carrying CFP at the amino terminal end, elicited a biphasic fluorescence change in which the quick fluorescence decrease due to DPA migration to the inner leaflet was followed by a slower additional quenching of the signal. Fitting the traces to a bi-exponential function indicated that this second component evolved with a time constant of 270 ± 22 ms (n = 18). The averaged voltage-dependent responses shown were not due to slow and irreversible photo-bleaching of the FP tag during the repetitive longer voltage steps used because: i) both the fast and the slow components returned to baseline and appeared readily reversed at the end of the depolarizing step, ii) the time course and magnitude of the averaged voltage-dependent responses during the voltage jumps remained the same when the number of delivered voltage steps was increased in some cells from the standard 50 up to 100 or 200 pulses, and iii) substituting the interrupted light protocol used for excitation (that prevented FP excitation during the interpulse intervals) by a continuous irradiation routine, yielded under our recording conditions a much slower, irreversible and essentially mono-exponential photo-bleaching decay of the FP signal, that developed with a time constant of 107 ± 18 s (n = 11). Altogether, our data suggest that the intramembrane translocation of the DPA is followed by some depolarization-dependent conformational rearrangement of the channel protein to which the FP is anchored, that reduces the orthogonal distance between the fluorophore located at the beginning of the amino terminus and the membrane surface. Interestingly, a similar trend was observed when the analysis was performed with a channel variant carrying the CFP donor at position 162, the second position along the sequence in which a FP tag was introduced and the hERG channel functionality was maintained^26^. Since residue 162 is located at the start of the highly flexible hERG proximal domain (Figs 1A and 4), this opens the possibility that, as discussed below, the re-allocation of the tag introduced at the amino terminal end may be influenced by some molecular reorganization(s) taking place in that region. Further work would be necessary to ascertain this hypothesis.

## Discussion

In this report we used a FRET-based approach to study the coarse topology of the hERG channel cytoplasmic domains in the native cell membrane. We used fully functional channels labeled at different positions with a FP tag acting as a FRET donor and DPA, a non–fluorescent absorber whose distribution switches between the inner and outer membrane leaflets according to the membrane potential, that is able to act as an acceptor for cyan, green and yellow FP variants^15–18,22–24^. We directly demonstrate for the first time that the distance between an amino terminal-located FP tag and the lipid bilayer, behaving as an independent reference plane, is much shorter than that between the membrane surface and a C-terminally-located label. Subsequent scanning of other channel attachment points at positions along the proximal domain (in the amino region) and the distal carboxy terminus, also demonstrated significant differences in the distance between the FP tags and the membrane, lying between those observed for the amino and carboxy terminal end-located labels. The existence of a long distance between the amino and carboxy end-located labels has been demonstrated by our previous measurements of FRET magnitudes between pairs of cyan and yellow FPs inserted in multiple sites along the hERG sequence^26^. However, since those estimations were obtained as relative distances between the labels, they were equally compatible both with a shorter distance between the amino end and the transmembranal core, and with an opposite upside-down organization of the cytoplasmic labels toward the membrane. It is important to note that our present measurements cannot allow for a quantitative estimation of absolute vertical distances, because the exact donor-acceptor ratio is unknown^20^. In the case of hERG, such measurements are further complicated by the poor plasma membrane versus intracellular distribution of the protein. In fact, only those channels expressed in the peripheral cell membrane will be affected by the voltage-dependent translocation of the DPA quencher in the voltage-clamped cells (ΔF), but different background fluorescence (affecting Fo) could change the ΔF/Fo ratio. However, independent of the absolute value of distance corresponding to the position of each construct, the different magnitude of the DPA-induced quenching is an excellent reporter of the relative distance of a given label toward the plane of the membrane, since in all cases an identical movement of DPA with respect to the fluorescent tag will take place in response to the same voltage jump. Note also that our data demonstrating that the same percentage of fluorescence signal exists in the plasma membrane as compared to that of the whole cell, when considering channels carrying the FP label in a different position (e.g. the amino and the carboxy terminal end) that exhibit a very divergent DPA-induced behaviour, unequivocally demonstrate that the observed ΔF/Fo differences are due to the different distances between the tag and the DPA (i.e. between the tag and the lipid bilayer plane in which the translocation of the DPA takes place). Therefore, aside from some methodological differences and variations in recording procedures used by different authors^15,16,19,23,24^, the different magnitude of the fast and voltage-dependent DPA-induced quench as recorded here, can be used to map the relative distance of a tag toward the membrane surface, given that it shows a definite spectral overlap with DPA.

A limitation of this experimental approach lies in the large size of the FP-based tags. Indeed, the distances considered here would correspond to those separating the FP chromophores in the centre of the barrel-like FP structure and the DPA, but not to those between the residues to which the labels are attached and the quencher. Despite these limitations, such measurements may contribute to better define the relative position of some channel domains with reference to each other and the membrane. The shortest distance was measured using the most N-terminally tagged construct, which is totally consistent with the proposed location of the hERG N-tail close to the transmembranal core^26,27,33–36^. However, it is important to note that the introduction of a fluorescent tag as needed for the DPA-dependent measurements makes it very unlikely that the native positioning of the N-tail embedded in a small cavity contacting the S4-S5 linker is maintained^10,37^. The fact that this construct shows accelerated deactivation kinetics as compared with the untagged channel^26^ further supports this interpretation. Interestingly, the fact that the amino terminal end FP label shows the closest positioning toward the membrane is further supported by the results obtained with the much smaller fluorophore FlAsH, whose emission spectrum is similar to YFP. We initially hypothesized that the reduced spectral overlap of FlAsH wih DPA as compared to that of CFP, leading to a lower DPA-induced quenching, could be compensated by a closer approximation of the small sized FlAsH to the membrane. However, only a slightly reduced level of quenching analogous to that observed with the YFP-1 labeled channel was obtained with the tetracysteine/FlAsH construct, that otherwise showed functional characteristics almost identical to those exhibited by the FP-labeled channel at the same position. This suggests that the presence of a voluminous YFP tag does not cause a greater structural disruption than FlAsH, and that the FlAsH tag-based labeling does not seem to draw the fluorophore closer to the membrane due to its smaller size. Thus, it is possible that the location of both labels is mainly determined by a global organization of the cytoplasmic domains instead of the nature and size of the tag. To the best of our knowledge, only an yellow YFP-like (or a red) variant of the FlAsH reagent is presently available. The very reduced level of quenching (around only 2%) obtained with FlAsH, even for the putatively closest-to-membrane labeled variant at position 1, indicates that the use of this type of reagent combined with DPA is severely limited by their small spectral overlap. Therefore, since the use of FlAsH did not allow for any improvement of the DPA-induced effects, and due to the easier technical handling of the FP-based methodology, this was routinely used in our experiments.

Apart from the demonstration that the FP tag inserted at the amino terminal end is located closest to the membrane, our data indicate that its positioning with respect to the plane of the bilayer changes in response to a constant and more maintained depolarization. Unfortunately, we have not yet been able to correlate the slow motion of the labels with any functional property. The need to track a large FP label, the increased capacitance added to the membrane by DPA itself and the convenience of using high amplitude voltage steps to saturate the DPA distribution at both sides of the bilayer, precluded a rigorous comparison with a specific gating characteristic. Nevertheless, since only a monophasic response was observed using an immobile donor such as GFP_f_, statically positioned toward the membrane, our results indicate that, at least under our technical conditions and time scale, the secondary and kinetically slow response is not due to delayed DPA transport across the membrane into the inner cytosolic space^17,20^. Furthermore, they strongly suggest that in response to more prolonged voltage steps, some depolarization-dependent conformational rearrangement(s) of the protein takes place that reduces the orthogonal distance of the fluorophore introduced at the amino terminus toward the membrane surface. It has been proposed that an extensive (and dynamic) network of interactions involving the N-terminal tail, the PAS domain and the highly flexible proximal domain of the amino terminus, the S4-S5 linker in the transmembranal core, and the C-linker and cNBD regions of the carboxy terminus, constitutes either an essential component of the hERG gating machinery or an important regulator of the gating process^27,37–45^. The formation of disulfide bridges between the amino terminal N-tail and some elements of the channel core and other cytoplasmic regions such as the S4-S5 and the C-linkers, sometimes dependent on the open or closed state of the channel, has been demonstrated^40–42^. Also, the participation of the N-tail on VSD to PD coupling has been repeatedly proposed for KCNH family members^27,44,46,47^, and the presence of this region seems to be essential for voltage-dependent potentiation (VDP, mode shift or hysteresis) to take place in these channels^45^. However, a direct proof of the N-terminus movement(s) has been lacking. The method used here is expected to be biased towards movements orthogonal to the membrane surface, but other conformational reorganizations parallel to the plane of the membrane cannot be excluded. Perhaps alternative approaches such as using unnatural fluorescent amino acids combined with VCF and FRET techniques^43,45,48–50^, could additionally yield important advances in this direction. It is tempting to speculate that the movements recognized here are part of the orchestrated interactions associated to the gating process. In this context, the reorganizations detected here can be related to some of such interactions and might modulate relevant aspects of channel gating. Interestingly, similar results were obtained with the CFP-162 construct, the most N-terminally cyan-labeled channel of our library of fluorescent hERG fusion proteins that maintains functionality^26^, also showing a sizeable DPA-induced quench. Apart from this being compatible with the highly flexible nature of the proximal domain extending from hERG residue 136 to 402 at the base of the S1 helix^10^, it opens up the possibility that the re-allocation of the tag at the amino terminal end could be related to some reorganization(s) in this domain. Our attempts to further confirm this hypothesis using a functional construct carrying a FP tag at residue 144, closer to the N-tail and PAS domain regions^26^, were hampered by its reduced level of expression, the availability of only a YFP-based variant at this position meaning a reduced spectral overlap with DPA and leading to a very reduced quenching, and the fact that, unlike hERG/CFP-162 that maintained gating properties similar to those of the wild-type channels, a strong positively shifted voltage dependence was observed with the YFP-144 construct (Suppl. Fig. 3).

The recent elucidation of the tridimensional structure of hERG and its relative eag1 channel by cryo-EM^10,51^ has provided an essential breakthrough in the understanding of possible differences with respect to other Kv channels and in achieving a greater knowledge about the molecular basis of their functional behaviour. However, some information related to the dynamic changes that seem to take place in the cytoplasmic domains during the complex chain of events associated with gating is still lacking. Noticeably, due to the need to biochemically stabilize the protein, the two reported hERG structures (hERG_T_ and hERG_Ts_) lack different sections of the proximal domain, and in both cases some changes in activation voltage dependence and conspicuous variations in closing kinetics are apparent^10^. Whereas the first alterations are coherent with the recognized impact of some proximal domain sections on activation kinetics^52,53^, both structures show the voltage sensing domains in depolarized conformation and with the intracellular gate open. Furthermore, in both cases the position of the N-tail at the beginning of the *eag*/PAS domain is the same. There is evidence that the correct positions of the hERG amino terminal structures determine the deactivation channel kinetics^26,27,33–36^. Therefore, the reason(s) for the much slower closing of the hERG_Ts_ variant^10^ are not directly evident from the structural data. Strikingly, a faster deactivation has been observed with other hERG proximal-deleted variants, in spite of the fact that they also show a similarly hyperpolarized activation voltage dependence^52,54^. Thus, it would be very interesting to know the exact positioning adopted by the amino terminal structures upon closing, their possible reorganizations along the gating processes and how this may depend on rearrangements of the flexible proximal domain, absent in the reported structures. The present data indicating the existence of some conformational reorganization of the amino terminal sections may provide some insight into this issue. Our initial attempts to detect DPA-dependent effects in amino terminally FP-labeled constructs combined with proximal domain-deleted channels proved unsuccessful. It remains to be established if the use of less voluminous tags also showing a more extensive spectral overlap with DPA, could improve this approach in the future.

## Methods

### Tagging of hERG channels

Generation of hERG channels either single or double N- and C-terminally labeled with the enhanced cyan (ECFP) or yellow (EYFP) fluorescent proteins (subsequently named CFP and YFP throughout the text) has been detailed elsewhere^26^. Constructs N- and C-terminally labeled with Cerulean were analogously obtained using pCerulean-C1 and pCerulean-N1 vectors (Addgene), respectively. We also used some members of our previously described collection of hERG channel constructs, in which a cyan (CFP) and/or yellow (YFP or VFP) label was introduced at different internal positions along the hERG sequence by random insertion with an *in vitro* transposition system (see ref.^19^). For fluorescein arsenical hairpin binder (FlAsH) tagging, a CCPGCC sequence was added to the N-terminal end of hERG generating a PCR fragment using a sense oligonucleotide containing a HindIII site, a Kozak’s signal, the start codon and the sequence for the CCPGCC residues, followed by the coding sequence for residues 2 to 10 of hERG together with the corresponding antisense oligonucleotide carrying the BstEII recognition site. The PCR fragment was digested with HindIII and BstEII, and subsequently used to replace the corresponding segment in hERG. A CCPGCC channel variant also carrying CFP at the carboxy terminus was routinely used for the experiments, in which the cyan fluorescence was used as a positive control to ensure an optimal level of channel expression in the cell elected for analysis and that an appropriate washout minimizing the FlAsH background of the nontransfected neighboring cells was achieved. The protein was subsequently labeled with the FlAsH biarsenical dye (Invitrogen) following the procedure detailed in ref.^32^. A labeling time of 1 h followed by 30 min of washout with 0.125 mM of the BAL reagent provided with the labeling kit, were used for optimal reduction of nonspecific binding while the specific labeling was maintained.

### Cell culture and transfection

Human embryonic kidney (HEK293) cells were grown at 37 °C in a humidified atmosphere of 95% air and 5% CO_2_ and plated in 35-mm diameter tissue culture plastic dishes containing sterile glass coverslips coated with polyL-lysine, that constituted the bottom of a recording chamber for electrophysiological and fluorometric measurements. The culture medium consisted of Dulbecco’s modified Eagle’s medium nutrient mixture F-12 Ham’s (DME/F12 1:1 mixture, Sigma and Lonza) supplemented with 100 U/ml penicillin, 0.1 mg/ml streptomycin and 10% fetal bovine serum. Cells trypsinized 24 h prior to transfection seeded on poly-L-lysine-coated coverslips were transiently transfected using Lipofectamine 2000 (Invitrogen) with 2–3 μg of plasmid DNA containing the different constructs. The mixture of DNA and Lipofectamine was incubated in serum-free medium for 20 min and added to the plates with the coverslips and the cells in serum-containing medium without antibiotics. The transfection reagent was removed 24 h later and complete culture medium was added. FlAsH labeling and/or recordings were typically performed 24–48 h after completion of transfection.

### Voltage-clamp fluorometry (VCF) in the presence of DPA

Recordings were performed at room temperature in the whole-cell configuration of the patch-clamp technique using a Zeiss Axiovert 100 microscope placed on top of a vibration isolation system (Newport, Irvine, CA, USA), with a 40×/0.75 Plan NeoFluar objective (Carl Zeiss). To improve the quality of the seals and the stability of the recordings, cells were sometimes mildly trypsinized and seeded on poly-L-lysine-coated coverslips to be used for analysis within 2–6 h. No change in the qualitative or quantitative characteristics of the cell currents or fluorometric recordings was introduced by this procedure. Patch pipettes with tip resistances of 2–4 MΩ were pulled from borosilicate disposable micropipettes (Boralex, Rochester Scientific, NY or Drummond Scientific Company, PA, USA). The standard extracellular saline contained (in mM): 137 NaCl, 4 KCl, 1.8 CaCl_2_, 1 MgCl_2_, 10 glucose, 10 HEPES (pH 7.4 with NaOH) and 10 μM DPA (Biotium) added immediately before recordings, when indicated. The pipette solution contained (in mM): 140 KCl, 2 MgCl_2_, 0.7 CaCl_2_, 1.1 EGTA, and 10 HEPES (pH 7.4 with KOH). An EPC-7 patch-clamp amplifier (HEKA Elektronics, Lambrecht, Germany) was used for voltage-clamp. Stimulation and electrophysiological or fluorometric data acquisition were performed with an ITC-16 ADC/DAC multichannel board (Instrutech Corp. NY) and the Pulse software (HEKA Elektronics) running on Macintosh computers. For data analysis the PulseFit (HEKA Elektronics) and IgorPro (WaveMetrics) software packages were used. The fluorescence system consisted of a monochromator Polychrome IV (Till Photonics, Gräfelfing, Germany) and a Till Photonic’s Photometric system equipped with a photomultiplier tube (Hamamatsu H5784 series), a control board for manual control of gain, and a ViewFinder III accessory to size and position the detection area on the microscope field, allowing for adjustment of this area overlayed to the transmission image of the sample on a video monitor and excluding from detection all surrounding areas not corresponding to the clamped cell. Synchronization of the voltage-clamp pulses and light measurements was achieved sending an analog voltage of the desired amplitude and duration to the Polychrome Manual Control (PMC) unit of the monochromator using the trigger routine of the Pulse software. According to the calibrations provided by the manufacturer, values of −1.35, +1.3 and +1.8 V were used to get 440, 498 and 510 nm wavelengths, to excite CFP, GFP and YFP/FlAsH, respectively. Even though in some cases these values did not correspond to the excitation maximum of the fluorophore, they were chosen to be optimally compatible with the tripleband 69008-ET-ECFP/EYFP/mCherry filter set (Chroma) incorporated to the microscope filter cube. Apart from providing enough excitation light to all the FP/FlAsH fluorophores and allowing for a substantial level of emission light collection, this set prevented continuous radiation of the samples with the 470 nm light coming from the monochromator, during the interpulse intervals in which the trigger output of the Pulse software remained at a resting value of zero volts. The readout of the photomultiplier was digitized at 5 KHz when short excitation pulses were used and at 1 KHz when long depolarization and voltage-clamp steps were applied. Averaged responses from 10–50 trials delivered at 1 or 2 s intervals, respectively, are shown on the graphs. ΔF/Fo versus V and I versus V relationships were fitted with a Boltzmann function to estimate the V_1/2_ and valence of the Boltzmann fit (*z*):

1 $${\rm{Y}}=1/[(1+\exp (({{\rm{V}}}_{1/2}-V)z{\rm{F}}/{\rm{R}}T)]$$

where *V* is the test potential and F, R and T are Faraday constant, gas constant and absolute temperature, respectively.

### Fluorescence microscopy of FP subcellular distribution

Images of cell fluorescence were obtained with the Zeiss Axiovert 100 microscope equipped with a Zeiss 100x oil-immersion TIRF objective (1.45 NA; Oil, Alpha Plan Fluar) and the Zeiss filter cube containing a polychroic mirror with reflection bands at 442 and 514 nm and band-passes at 475/30 and 560/60 nm (z442/514/633rpc), and two z442/514x and z440/514 m excitation and emission filters (Chroma), respectively. YFP fluorescent emission at 515 nm excitation wavelength was collected and processed with an Imago 12-bit CCD camera (Till-Photonics). Images were acquired without binning at 1376 × 1040 maximal resolution of the camera. The camera and monochromator were controlled by TILLvisION 4.0 software, that was also used for image recording and processing. The objective was attached to a piezo focusing device controlled by the TILLvisION software, and used to acquire a *z* stack series of the fluorescence images. Election of the best in focus image of the series was subsequently performed either by eye or using the AutoFocus function of the software.

### Statistics

Data values given in the text and in figures with error bars represent the mean ± SEM for the number of indicated cells. Comparisons between data groups were at first performed by parametric Student’s impaired *t* test (2-tailed). When significant differences in standard deviation were present an alternate Welch’s test or non-parametric Wilcoxon or Mann-Whitney test were also used. In all cases, *p*-values < 0.05 were considered as indicative of statistical significance.

## Electronic supplementary material

Supplementary Information
