# Supplementary material for: Relative positioning of Kv11.1 (hERG) K+ channel cytoplasmic domain-located fluorescent tags toward the plasma membrane
Source: Sci Rep. 2018 Oct 19;8:15494. doi: 10.1038/s41598-018-33492-x (PMC6195548; doi:10.1038/s41598-018-33492-x)
Supplement: Supplementary file 1 — Supplementary Information [file 41598_2018_33492_MOESM1_ESM.doc]

**SUPPLEMENTARY INFORMATION**

Relative positioning of Kv11.1 (hERG) K^+^ channel cytoplasmic domain-located fluorescent tags toward the plasma membrane

Francisco Barros*, Pedro Domínguez and Pilar de la Peña


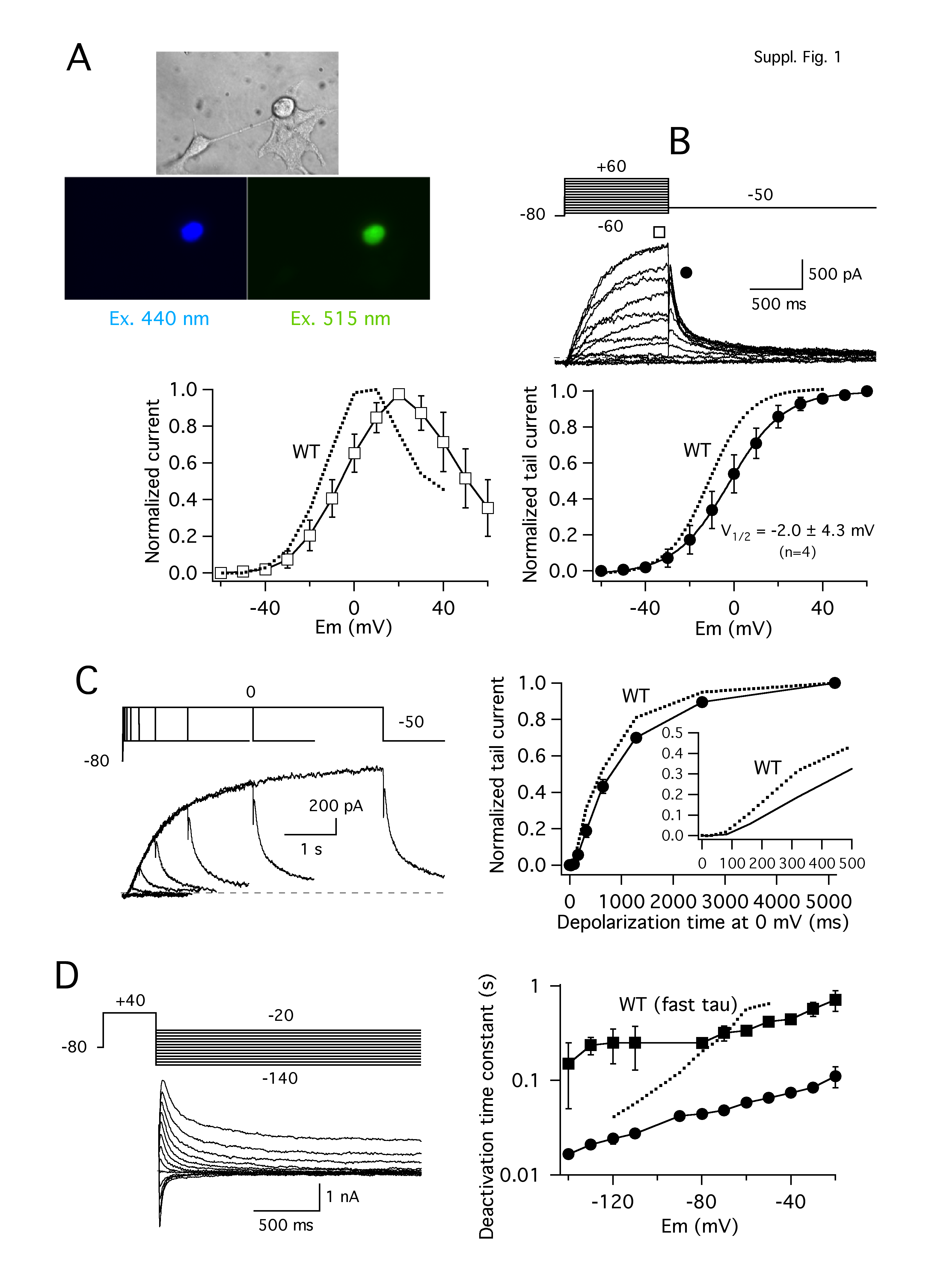


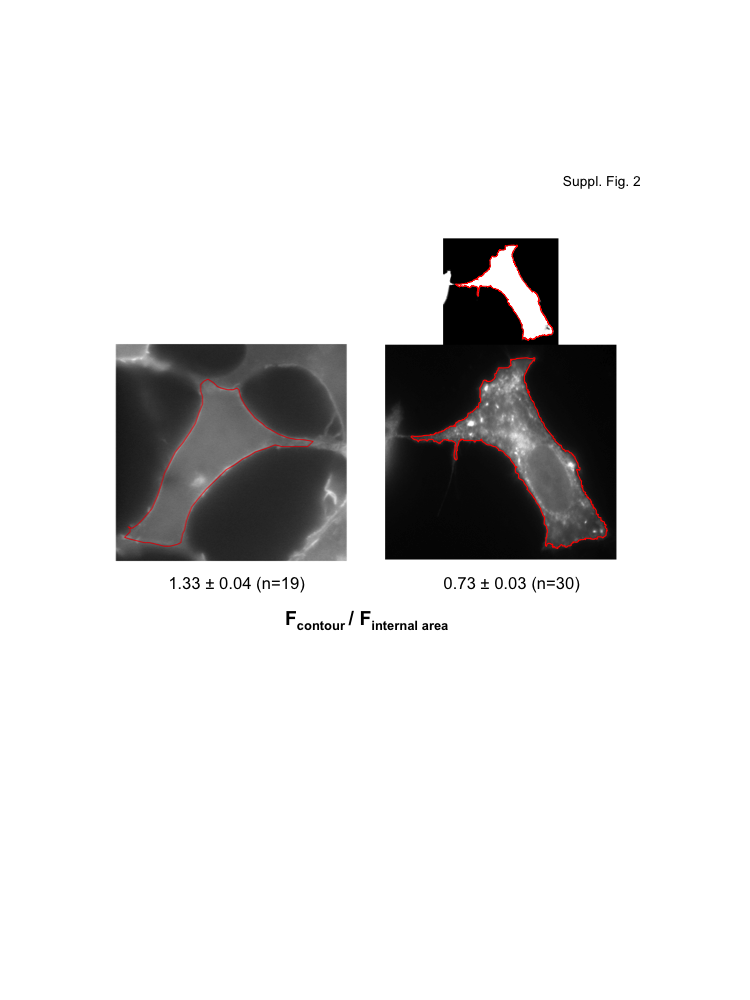


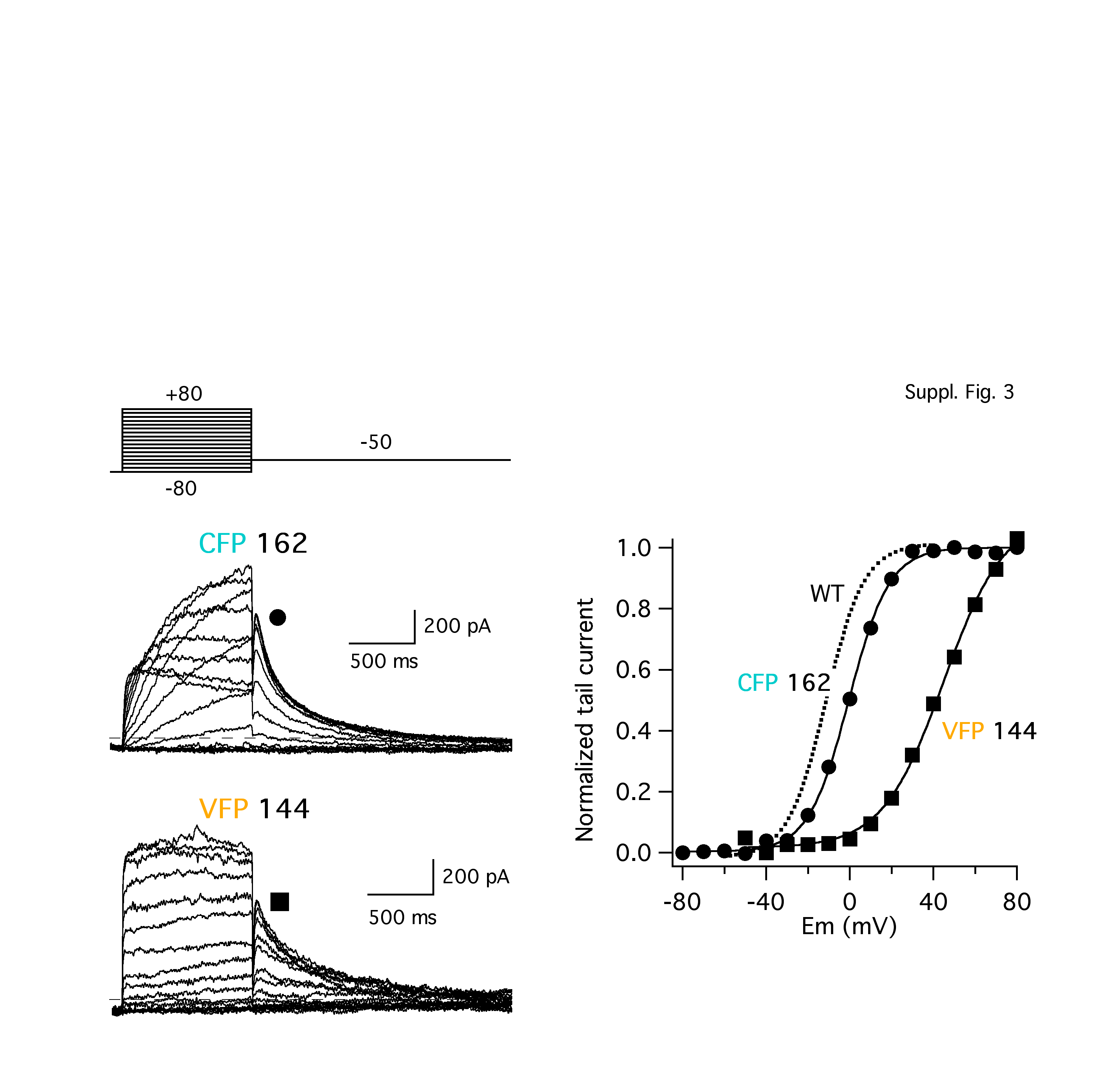


**SUPPLEMENTARY FIGURE LEGENDS**

**Supplementary Fig. 1.** Demonstration of specific labeling with FlAsH of cells expressing hERG channels carrying a CCPGCC sequence at the amino terminus and CFP at the carboxy end, and electrophysiological characterization of the tagged channels. **A.** A transmission image of the microscope field (40x objective) is represented at the top. Fluorescence images of the same field obtained at excitation wavelengths of 440 (CFP fluorescence, left) and 515 nm (FlAsH fluorescence, right) are shown at the bottom. Note the high fluorescence intensity of the FlAsH-labeled cell also showing a high level of CFP fluorescence, as compared with the neighboring cells non-efficiently transfected with the CFP-tagged channel. **B.** Activation voltage dependence of the tetracysteine/FlAsH-tagged construct. A representative family of currents from a cell submitted to 1-s depolarization pulses at different potentials and at 10 mV intervals from a holding potential of -80 mV, followed by a repolarization step at -50 mV, is shown at the top. Note the considerably fast decay of the tail currents at -50 mV. Averaged *I* versus *V* relationships normalized to maximum, measured at the end of the depolarization step (open square) and at the peak of the tail current at -50 mV (closed circle) are shown at the bottom. The continuous line in the normalized tail *vs.* voltage plot is a Boltzmann fit to the data. Values from untagged wild-type channels (WT) are shown as dotted lines for comparison. **C.** Voltage-dependent activation rate at 0 mV. A family of representative membrane currents from a cell submitted to depolarizing prepulses to 0 mV of variable duration, followed by a repolarizing step to -50 mV, is shown on the left. An averaged plot of normalized tail current magnitude versus depolarization time is depicted on the right. An expansion of the initial 500 ms to highlight the early current delay in the sigmoidal activation time course is shown in the inset. Values from untagged wild-type channels are shown as dotted lines for comparison. **D.** Accelerated voltage-dependent deactivation kinetics in the tetracysteine/FlAsH-tagged channels. A representative family of currents is shown on the left, obtained during steps to potentials ranging from -20 to -140 mV in 10 mV intervals, following depolarization pulses at +40 mV to open (and inactivate) the channels, using the indicated protocol. The dependence of deactivation rates on repolarization membrane potential is shown on the right. Deactivation time constants were quantified by fitting a double exponential to the decaying portion of the tails. The magnitude of the deactivation time constant corresponding to the fast decaying component, obtained from untagged wild-type channels, is shown as a dotted line for comparison.

**Supplementary Fig. 2.** Quantitative comparison of fluorescence intensity levels in the cell contour and the internal cell area. The image on the left shows an enhanced view of a HEK293 cell expressing the thyrotropin-releasing hormone (TRH) receptor (a protein exhibiting a major subcellular distribution along the cell plasma membrane; see ref. 19) carrying YFP attached to the carboxy end. A red line following the cell contour generated manually using the brightest illuminated pixels at the edge of the cell, that was used to quantify the averaged fluorescence of the image pixels corresponding to the plasma membrane, is shown superimposed. The value shown at the bottom indicates the fluorescence intensity ratio obtained dividing the averaged fluorescence intensity of the pixels coming from the line on top of the cell edge with respect to the averaged fluorescence in the internal cell area. In this second case, fluorescence levels were estimated generating a region of interest (ROI) with the same shape as the contour line but sized around 95% of it and measuring the averaged fluorescence intensity from the whole area delimited by the ROI. Numbers correspond to mean ± S.E. for the indicated cells from three independent transfections. Note the F_contour_/F_internal area_ value analogous to that obtained upon expression of Rho-pYC in Fig. 5. Images for an equivalent analysis corresponding to a cell expressing hERG WT channels labeled with YFP at position 1 are depicted on the right. Due to the dim plasma membrane perimeter obtained with the hERG-expressed constructs, in this case the cell contour was defined employing an over-exposed image as shown at the top and using the generated mask and its contour line defining the edge of the cell to obtain the F_contour_/F_internal area_ ratio (0.73 ± 0.03, n=30) as indicated above.

**Supplementary Fig. 3.** Comparison of voltage-dependent activation properties of FP-tagged channels at positions 144 and 162. Representative families of currents from cells submitted to 1-s depolarization pulses at different potentials and at 10 mV intervals from a holding potential of -80 mV, followed by a repolarization step at -50 mV, are shown on the left. Averaged *I* versus *V* relationships normalized to maximum, measured at the peak of the tail currents at -50 mV are shown on the right. The continuous lines in the normalized tail *vs.* voltage plots are Boltzmann fits to the data. A curve from untagged wild-type channels (WT) is shown as a dotted line for comparison. Note the prominent decay of the current magnitudes during the depolarization steps at very positive voltages in the case of the 162-tagged channels, due to the slow activation and fast inactivation overlap that leads to the typical inward rectification exhibited by the wild-type hERG channels, and the considerably altered kinetic properties of the 144-tagged variant.
